# Cognitive and cerebral perfusion outcomes following interventional revascularization in individuals with chronic middle cerebral artery occlusion

**DOI:** 10.3389/fneur.2025.1667246

**Published:** 2025-11-07

**Authors:** Liang Yin, Pingli Sui, Qianqian Li, Juanjuan Xu, Jiali Xu, Xianwen Chen

**Affiliations:** 1 Department of Neurology, The First Affiliated Hospital of Anhui Medical University, Hefei, China; 2 Department of Neurology, The First Affiliated Hospital of Bengbu Medical University, Bengbu, China; 3 Department of Imaging, The First Affiliated Hospital of Bengbu Medical University, Bengbu, China

**Keywords:** cerebral perfusion parameters, chronic MCAO, cognitive impairment, interventional surgery, MOCA score

## Abstract

**Background:**

Chronic occlusion of the middle cerebral artery (MCAO) is frequently underdiagnosed due to mild or insidious clinical manifestations. Despite this, individuals with chronic MCAO are at an increased risk of cerebral infarction and progressive cognitive impairment. This study aimed to assess the efficacy of interventional revascularization therapy in comparison to standard medical treatment, with a focus on neurological and cognitive outcomes.

**Methods:**

Fifty-five patients diagnosed with chronic MCAO were allocated to either a medication group or an interventional surgery group. Neurological status and cerebral perfusion parameters, including cerebral blood flow (CBF), time-to-maximum (Tmax), mean transit time (MTT), and cerebral blood volume (CBV) were assessed. Cognitive performance was assessed using the Montreal Cognitive Assessment (MoCA) and Mini-Mental State Examination (MMSE). Post-treatment and follow-up data were collected and analyzed.

**Results:**

Among the 29 participants in the interventional surgery group, successful recanalization was achieved in 26 cases, while three procedures were unsuccessful. Improvements in neurological deficits were observed in both groups. However, significant enhancements in cerebral perfusion parameters (CBF, Tmax, and MTT) and cognitive scores (MoCA and MMSE) were identified only in the interventional group. No statistically significant changes in cerebral perfusion or cognitive performance were observed in the medical therapy group.

**Conclusion:**

Interventional revascularization therapy was associated with improved cerebral perfusion and enhanced cognitive outcomes compared to medical management in individuals with chronic MCAO.

## 1 Background

Ischemic stroke represents a major public health concern due to its high rates of mortality and long-term disability. In China, stroke-related mortality significantly exceeds that associated with other conditions, accounting for approximately one-third of global stroke-related deaths (1). Occlusion of the middle cerebral artery (MCAO) is a principal etiological factor in ischemic stroke. Among individuals with stroke in Asian populations, the incidence of intracranial large artery occlusion has been reported to reach 34.5% (2). Chronic MCAO often remains undiagnosed due to its subtle clinical presentation.

Unlike acute ischemic stroke, which typically manifests with marked neurological deficits, patients with chronic MCAO may not exhibit overt neurological symptoms. However, findings have indicated that chronic MCAO was closely associated with ischemic events, contributing to approximately 10% of ischemic strokes. Reported stroke risk in this population ranges from 3.6% to 22.0%, and recurrence occurs in 23.4% of patients despite optimal medical therapy (3, 4).

Prolonged MCAO leads to chronic cerebral hypoperfusion in brain regions distal to the occlusion, contributing to subclinical cerebral injury through pathophysiological mechanisms distinct from those of acute infarction (5). Even in the absence of overt ischemic symptoms, chronic reductions in perfusion may lead to progressive brain atrophy, manifesting as cognitive decline (6). Consequently, individuals with intracranial arterial occlusion are at risk for cognitive decline secondary to chronic hypoperfusion. In more advanced stages, neurological deficits may become clinically apparent, potentially leading to motor dysfunction, reduced independence in activities of daily living, or mortality (7, 8). The risk of recurrent stroke also remains elevated in this population (9).

Historically, the primary approach to managing chronic MCAO has involved pharmacological therapy. However, this strategy does not address the underlying vascular occlusion or the associated hemodynamic insufficiency (10, 11). Consequently, patients managed solely with medical therapy remain vulnerable to subsequent ischemic events (4). Interventional revascularization offers the potential to restore perfusion to downstream brain tissue, thereby enhancing cerebral metabolism and potentially attenuating or reversing cognitive deterioration. Despite these theoretical advantages, available research on the efficacy of interventional procedures for chronic MCAO remains limited (12). Existing investigations have predominantly focused on the alleviation of postoperative neurological deficits, with relatively little focus on cognitive recovery.

The present study employed neuroimaging techniques and cognitive assessments to evaluate neurological status and cognitive outcomes in patients with chronic MCAO. Comparisons were made between those receiving medical therapy and those undergoing interventional revascularization to assess differences in cerebral perfusion and cognitive recovery. These findings were intended to contribute to the growing body of evidence regarding the potential cognitive benefits of endovascular revascularization in this population.

## 2 Methods

### 2.1 Study participants

A total of 55 patients diagnosed with chronic MCAO at Bengbu Medical College between November 2019 and November 2024 were enrolled in this study. The cohort was randomly divided into two groups: 26 patients received interventional surgery in combination with medication, while 29 patients received medication alone. For the subsequent assessment of the patient's neurological and cognitive functions, the group information of the patients is unknown to the evaluators. All patients underwent cranial magnetic resonance imaging (MRI) or computed tomography (CT), in addition to CT angiography (CTA) of the cerebral and cervical arteries. Diagnostic confirmation required evidence of M1 segment occlusion of the middle cerebral artery by digital subtraction angiography (DSA), absence of significant neurological deficits or the presence of only mild to moderate chronic ischemic symptoms, and clinical stability at the time of enrollment. The chronic nature of the occlusion was defined as a duration exceeding 4 weeks, determined based on the patient's symptom history (13).

Inclusion criteria: (1) Unilateral MCAO confirmed by DSA with clinical manifestations such as transient ischemic attack (TIA) or mild stroke; if additional cerebral artery stenoses were present, the degree of stenosis was < 50%; (2) CT perfusion (CTP) imaging indicating decreased cerebral blood flow (CBF) and evidence of hemodynamic compromise in the vascular territory distal to the occlusion; (3) Occlusion located proximal to the bifurcation of the M1 segment of the MCA, with retrograde collateral filling of the bifurcation or distal branches via meningeal branches of the anterior cerebral artery, and an American Society of Interventional and Therapeutic Neuroradiology/Society of Interventional Radiology (ASITN/SIR) collateral flow grade > 3; (4) Absence of major neurological deficits, with symptoms limited to mild to moderate impairment such as TIA, mild hemiparesis, sensory abnormalities, or cognitive decline, persisting stably for > 4 weeks, and a modified Rankin Scale (mRS) score ≤ 2; (5) Provision of informed consent by both the patient and their family; (6) Absence of vascular tortuosity that would hinder endovascular access to the occlusion site.

Exclusion criteria: (1) MCA occlusion secondary to Moyamoya disease, arterial dissection, arteritis, or occlusion of the internal carotid artery or other large intracranial arteries, or the presence of severe stenosis; (2) Absence of a clearly defined cerebral hypoperfusion zone on CTP imaging; (3) Presence of severe systemic disease or significant neurological impairment, defined as mRS score > 3; (4) History of infarction affecting critical cognitive regions, presence of extensive white matter lesions, Alzheimer's disease, or other cognitive disorders; (5) Refusal of treatment by the patient and/or their family.

### 2.2 Neurological function assessment

#### 2.2.1 National Institutes of Health Stroke Scale (NIHSS) score

Neurological deficits were evaluated using the NIHSS, which was administered by trained physicians. Total scores range from 0 to 42, with higher scores indicating greater severity of neurological dysfunction.

#### 2.2.2 Modified Rankin Scale (mRS) score

Functional status and disability levels were evaluated using the modified Rankin Scale (mRS), administered by neurologists or trained assessors according to standardized procedures. The mRS scoring system ranges from 0 to 5: 0: No symptoms; 1: No significant disability; symptoms present but not affecting daily activities; 2: Slight disability, independent in daily activities despite some limitations; 3: Moderate disability, able to of walk unassisted but requiring some support for daily tasks; 4: Moderately severe disability, unable to perform daily activities independently; 5: Severe disability, requiring continuous care.

### 2.3 Treatment methods

#### 2.3.1 Pharmacological treatment

All participants, regardless of group assignment, received pharmacological therapy consisting of aspirin (100 mg), clopidogrel (75 mg), and rosuvastatin (10 mg), each administered once daily over a 6-month period.

#### 2.3.2 Interventional surgical treatment

Participants assigned to the surgical group underwent endovascular treatment under either general or local anesthesia, as per individual clinical circumstances. For femoral artery access, an 8F guiding catheter in combination with a 6F intermediate catheter was used. In cases involving radial artery access, a 6F intermediate catheter was employed exclusively. A 0.035-inch hydrophilic guidewire was used to navigate the intermediate catheter into the internal carotid artery near the cavernous sinus. A microcatheter and a 0.014-inch micro-guidewire was advanced through the occlusion site. Following angiographic confirmation of intraluminal positioning, balloon dilation was performed to reduce luminal stenosis to < 10%. If no dissection was detected, angiography was repeated after a 20-min interval. In the absence of significant recoil, the procedure was considered complete. If vessel recoil or dissection-like changes were observed, a self-expanding or balloon-expandable stent was deployed based on vascular morphology.

Surgical success was defined as achieving either residual stenosis < 10% or a Thrombolysis in Cerebral Infarction (TICI) grade > 2b. Dual antiplatelet therapy with aspirin (100 mg) and clopidogrel (75 mg) was administered for at least 7 days prior to the procedure. Postoperatively, blood pressure was closely monitored to minimize the risk of reperfusion injury and intracerebral hemorrhage. Aspirin, clopidogrel, and rosuvastatin were continued for 6 months. A cranial CT scan was performed immediately after surgery to exclude intracranial hemorrhage. Management of vascular risk factors continued after the procedure, with guidance provided to support healthy lifestyle maintenance.

### 2.4 DSA

Whole-brain DSA was performed preoperatively and repeated 6 months postoperatively. Preoperative evaluations included assessment of the morphology of the occlusion stump, the length of the occluded segment, the distal endpoint of the lesion, the presence of diffuse vascular pathology beyond the occluded area, and the status of collateral circulation as graded by the ASITN/SIR scale. Immediate postoperative cerebral perfusion was evaluated using the TICI grading system. At the 6-month follow-up, DSA was employed to evaluate vascular patency and detect evidence of restenosis.

ASITN/SIR collateral grading criteria: Grade 0: No collateral perfusion to the ischemic region; Grade 1: Slow collateral perfusion reaching the periphery of the ischemic zone; Grade 2: Rapid collateral perfusion partially reaching the ischemic region; Grade 3: Slow but complete perfusion of the ischemic area during the late venous phase; Grade 4: Rapid and complete retrograde collateral perfusion of the entire ischemic territory.

TICI grading criteria: Grade 0: No perfusion beyond the site of occlusion; Grade 1: Minimal contrast penetration beyond the occlusion without distal vessel perfusion; Grade 2a: Partial perfusion with delayed filling and clearance in less than two-thirds of the affected territory; Grade 2b: Partial perfusion with delayed clearance but complete filling of distal vessels; Grade 3: Complete and rapid perfusion of distal segments (M3 and M4) with normal clearance.

### 2.5 Cerebral perfusion parameter measurement

All participants underwent cranial CT and whole-brain CTP imaging using a 256-slice Revolution CT scanner (GE Healthcare, USA). Imaging protocols employed a slice thickness and interslice spacing of 5 mm. CTP data acquisition was performed using a 16 cm wide-area detector, with the following parameters: gantry rotation time of 0.28 s, tube voltage of 80 kV, tube current of 150 mAs, and a display field of view (DFOV) of 25 × 35 cm. The ASiR-V reconstruction algorithm was utilized. Intravenous contrast administration involved an initial injection of 20 mL 0.9% sodium chloride via the antecubital vein, followed by rapid injection of 50 mL iodixanol contrast agent (320 mgI/mL) at 5 mL/s, and a subsequent 40 mL saline flush. Image acquisition commenced 5 s after contrast injection, capturing 20 image sets at 2-s intervals. All imaging data were transferred to the GE AW4.7 workstation. Using the CT Perfusion4D software and a deconvolution algorithm, pseudocolor maps of CBF, cerebral blood volume (CBV), mean transit time (MTT), and time-to-maximum (Tmax) were generated. For each patient, the axial slice demonstrating the greatest reduction in perfusion on the Tmax map was selected. A circular region of interest (ROI) was placed within the area of hypoperfusion to enable quantitative analysis of perfusion parameters.

### 2.6 Cognitive function assessment

Cognitive performance was evaluated using the Montreal Cognitive Assessment (MoCA) and the Mini-Mental State Examination (MMSE) at baseline (day of hospital admission) and at 6 months following either pharmacological or interventional treatment.

#### 2.6.1 MoCA assessment

The MoCA is a 30-point screening tool designed to evaluate various cognitive domains. For patients with ≤ 12 years of education, one point was added to the total score, with a maximum possible score of 30. Each assessment was completed within 10 min by neurologists or trained evaluators using standardized protocols.

#### 2.6.2 MMSE assessment

The MMSE is a 30-point cognitive screening instrument. A score >17 is considered normal for illiterate individuals, >20 for those with < 6 years of education, and >24 for those with >6 years of education. Evaluations were completed within 10 min by neurologists or trained professional staff according to standardized procedures.

#### 2.7 Statistical analysis

All statistical analyses were conducted using SPSS version 19.0, and data visualization was performed using GraphPad Prism version 8.0. Data conforming to normal distribution are presented as mean ± standard deviation (Mean ± SD). Between-group comparisons were performed using Student's *t*-test. Categorical variables were analyzed using the chi-square test. Pearson correlation analysis was employed to examine associations between continuous variables. A two-tailed *p*-value < 0.05 was considered to indicate statistical significance.

## 3 Results

### 3.1 Demographic and clinical characteristics

A total of 55 patients diagnosed with chronic MCAO were enrolled and assigned to either the medication group (*n* = 29) or the interventional surgery group (*n* = 26). Among these, 21 patients presented with left-sided MCAO, while 24 exhibited right-sided MCAO. Among the patients in medication group, the proportion of left-sided involvement was 34.62%, while for the patients in the interventional surgery group was 42.31%. Baseline mRS scores were as follows: 10 patients scored 0, 27 scored 1, and 18 scored 2. As presented in Table 1, there were no statistically significant differences between the two groups regarding baseline demographic or clinical characteristics, including age, sex, hypertension, diabetes mellitus, coronary artery disease, tobacco use, alcohol consumption, total cholesterol, triglyceride levels, or platelet count.

**Table 1:** Baseline demographic and clinical characteristics of patients with chronic middle cerebral artery occlusion.

**Clinical Characteristics**	**Patients accepted drug (*n* = 29)**	**Patients accepted surgery (*n* = 26)**	***P*-value**
Ages	59.10 ± 13.30	64 ± 32.91	0.206
**Gender**			
Male	22	20	
Female	7	6	0.926
**Hypertension**			
Yes	11	12	
No	18	14	0.537
**Diabetes**			
Yes	10	6	
No	19	20	0.352
**CHD**			
Yes	4	1	
No	25	25	0.417
**Smoking**			
Yes	9	9	
No	20	17	0.778
**Drinking**			
Yes	7	4	
No	22	22	0.418
Total cholesterol	3.93 ± 0.69	3.91 ± 1.05	0.887
Triglyceride	1.90 ± 1.05	1.65 ± 0.85	0.341
Soterocyte	213.55 ± 50.58	214.69 ± 52.53	0.935

### 3.2 Treatment process and vascular follow-up

**Figure 1:**
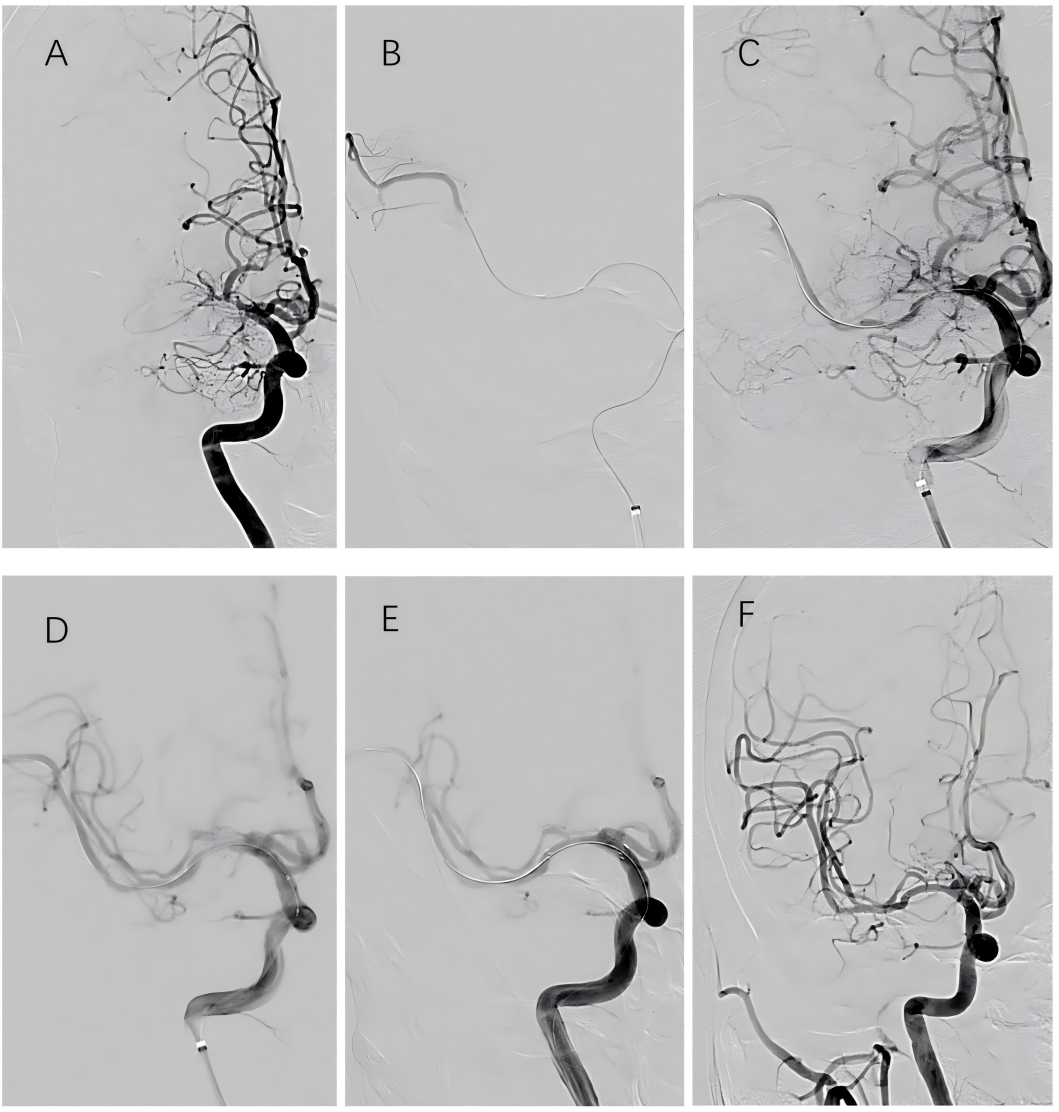
Endovascular procedure for middle cerebral artery recanalization. **(A)** Angiography via intermediate catheter showing occlusion at the MCA origin. **(B)** Microcatheter and microguidewire advanced through the occlusion; with angiographic confirmation of true lumen access. **(C)** Microcatheter withdrawn while retaining microguidewire position. **(D)** Balloon angioplasty performed, showing partial reopening with residual stenosis. **(E)** Deployment of a self-expanding stent resulting in vessel patency and elimination of residual stenosis. **(F)** Final angiography following guidewire removal demonstrating complete recanalization.

**Figure 2:**
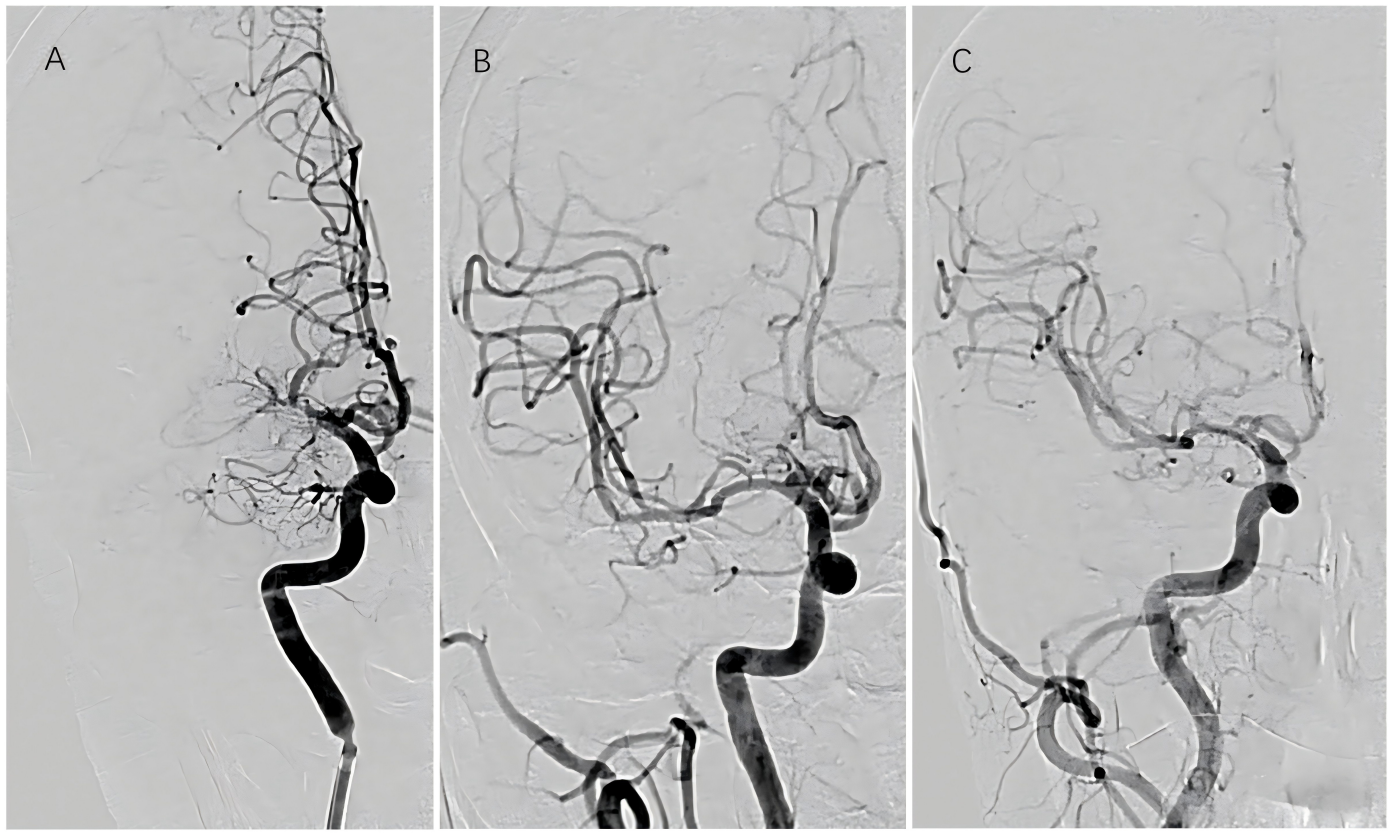
Sequential vascular imaging of recanalized segment. **(A)** Preoperative angiography; **(B)** immediate postoperative angiography; **(C)** angiographic evaluation at 6-month follow-up.

Among the 29 patients who underwent interventional surgery, successful recanalization was achieved in 26 cases, while three procedures were deemed unsuccessful. All patients who underwent successful recanalization demonstrated residual stenosis of < 10% immediately postoperatively. According to the TICI grading system, four patients achieved grade 2b perfusion and 22 achieved grade 3 perfusion. Recanalization was achieved in 11 cases of left MCAO and 15 cases of right MCAO. Of the surgical cases, balloon angioplasty alone was performed in 13 patients, while 13 required stent implantation (nine with self-expanding stents and four with balloon-expandable stents). No cases of intraoperative hemorrhage were observed. Immediate postoperative cranial CT identified minor contrast extravasation in five patients; however, no worsening of neurological deficits occurred. Perforator-related complications were documented in four cases, including dysarthria (*n* = 1), limb numbness (*n* = 2), and decreased muscle strength (*n* = 1), all of whom recovered satisfactorily. No embolic events were reported. At the 6-month follow-up, two patients developed new mild cerebral infarctions: one due to re-occlusion and one secondary to a perforator-related event. Additionally, three patients exhibited > 50% restenosis (Figures 1, 2). In the medication group, three patients experienced new cerebral infarctions during the follow-up period, all categorized as minor strokes. One additional patient experienced a TIA, which was effectively managed with pharmacological treatment.

### 3.3 Cerebral perfusion parameters

**Figure 3:**
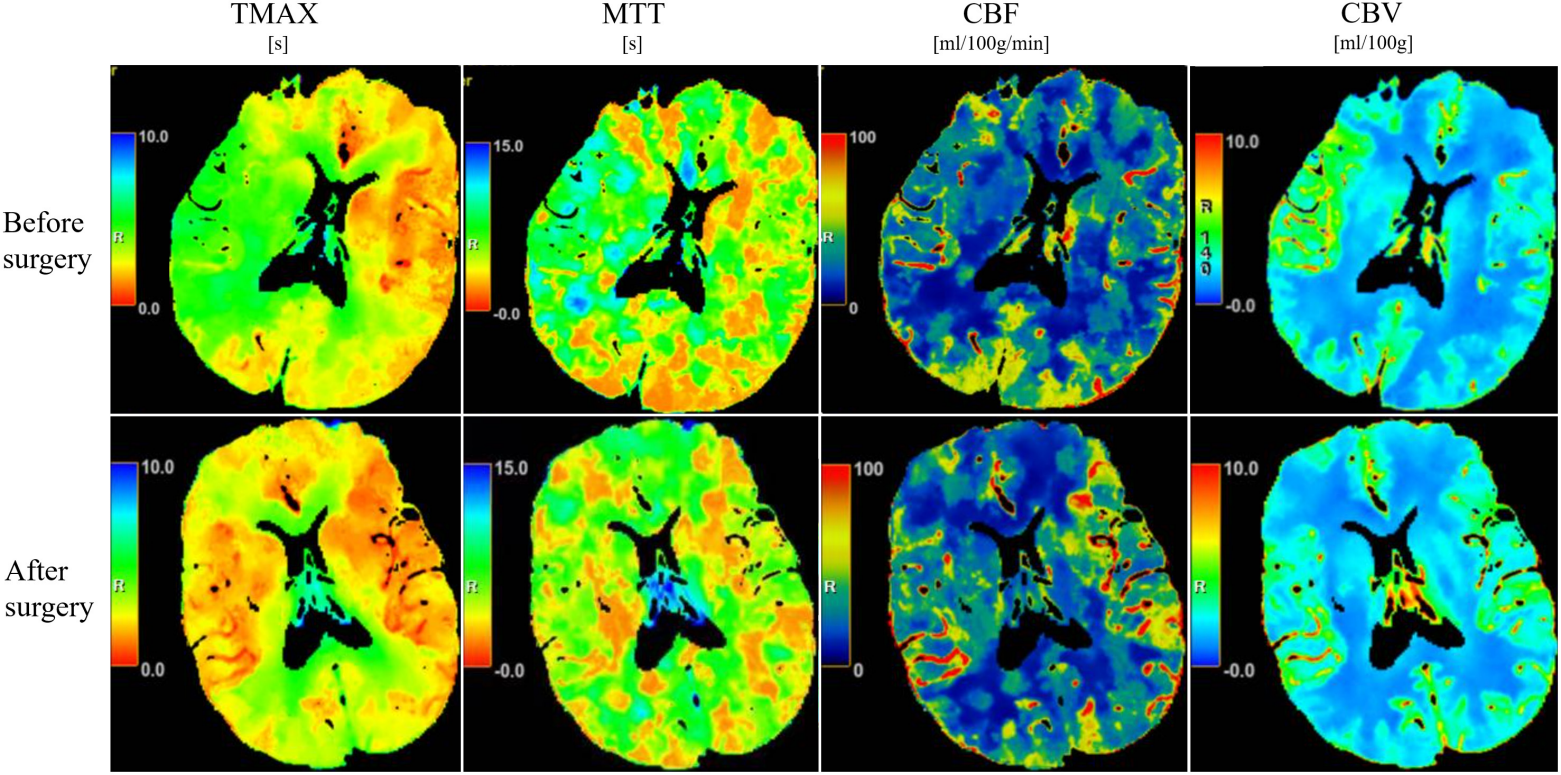
CT perfusion imaging before and after surgical intervention. Pseudocolor maps illustrating changes in Tmax, MTT, CBF, and CBV in a representative patient treated with combined endovascular and pharmacological therapy.

No statistically significant changes were observed in cerebral perfusion parameters—including CBF, CBV, Tmax, and MTT—in the medication group following treatment (*p* > 0.05). In contrast, patients in the interventional surgery group exhibited large ischemic regions in the right MCA territory on preoperative Tmax and MTT maps, which showed marked resolution at 6 months postoperatively (Figure 3). Quantitative analysis showed significant improvements in perfusion parameters following intervention: CBF increased from 24.42 ± 4.69 to 40.91 ± 7.62 (*p* < 0.001), Tmax decreased from 5.19 ± 1.23 to 2.82 ± 0.52 (*p* < 0.001), and MTT decreased from 7.70 ± 1.24 to 4.86 ± 0.79 (*p* < 0.001) (Table 2; Figure 3). These findings indicate that interventional surgery yielded superior improvements in cerebral hemodynamics compared with pharmacological treatment alone.

**Table 2:** Comparison of cerebral perfusion parameters before and after treatment.

**Perfusion parameters**	**Patients accepted drug (*****n*** = **29)**			**Patients accepted surgery (*****n*** = **26)**		
	**Before drug**	**After drug**	***P*** **-value**	**Before surgery**	**After surgery**	***P-*** **value**
CBF	22.59 ± 2.66	24.15 ± 3.91	0.08	24.42 ± 4.69	40.91 ± 7.62	< 0.001^*^
CBV	2.46 ± 0.37	2.33 ± 0.72	0.40	2.49 ± 0.37	2.48 ± 0.52	0.96
Tmax	5.43 ± 1.19	4.91 ± 1.44	0.14	5.19 ± 1.23	2.82 ± 0.52	< 0.001^*^
MTT	7.82 ± 1.30	7.38 ± 0.13	0.13	7.70 ± 1.24	4.86 ± 0.79	< 0.001^*^

^*^P < 0.05.

### 3.4 Neurological function scores

Post-treatment evaluations demonstrated significant improvement in neurological function in both groups. In the medication group, the NIHSS score decreased from 2.45 ± 1.53 to 1.62 ± 1.05 (*p* < 0.05), and the mRS score decreased from 2.03 ± 1.09 to 1.45 ± 0.87 (*p* < 0.05). In the interventional surgery group, NIHSS scores declined from 1.81 ± 1.52 to 0.73 ± 0.78 (*p* < 0.01), and mRS scores from 2.15 ± 1.01 to 1.35 ± 0.94 (*p* < 0.01). While both treatment modalities improved neurological outcomes, the extent of improvement was significantly greater in the interventional group (Table 3).

**Table 3:** Changes in neurological function scores before and after treatment.

**Scoring tools**	**Patients accepted drug (*****n*** = **29)**			**Patients accepted surgery (*****n*** = **26)**		
	**Before drug**	**After drug**	***P-*** **value**	**Before surgery**	**After surgery**	***P-*** **value**
NISS	2.45 ± 1.53	1.62 ± 1.05	0.019^*^	1.81 ± 1.52	0.73 ± 0.78	0.002^*^
mRS	2.03 ± 1.09	1.45 ± 0.87	0.027^*^	2.15 ± 1.01	1.35 ± 0.94	0.004^*^

^*^P < 0.05.

### 3.5 Cognitive function scores

In the interventional surgery group, MoCA total scores increased significantly from 18.65 ± 2.51 to 21.58 ± 2.04 (*p* < 0.001). Improvements were particularly pronounced in memory (3.31 ± 0.93 to 3.81 ± 0.85, *p* < 0.05), attention (3.69 ± 0.88 to 4.19 ± 0.85, *p* < 0.05), and abstract reasoning (1.00 ± 0.69 to 1.42 ± 0.81, *p* < 0.05). No statistically significant changes were observed in the visuospatial/executive, naming, language, or orientation subdomains (all *p* > 0.05). In contrast, the medication group demonstrated no significant improvement in either the overall MoCA score or individual subdomain scores after treatment (Table 4). For MMSE scores, no statistically significant change was observed in the medication group (22.72 ± 1.98 vs. 23.86 ± 2.34, *p* = 0.051), whereas the interventional surgery group demonstrated a significant increase from 23.12 ± 2.89 to 25.62 ± 2.35 (*p* < 0.05) (Table 4). These results indicate that interventional revascularization was associated with greater improvements in cognitive function compared to medication alone among patients with chronic MCAO.

**Table 4:** Changes in cognitive function scores before and after treatment.

**Scoringtools**	**Patients accepted drug**			**Patients accepted surgery**		
	**Before drug**	**After drug**	***P-*** **value**	**Before surgery**	**After surgery**	***P-*** **value**
MoCA	19.17 ± 2.61	20.38 ± 2.82	0.09	18.65 ± 2.51	21.58 ± 2.04	< 0.001^*^
Visual space	3.66 ± 0.67	3.72 ± 0.75	0.71	3.04 ± 0.92	3.54 ± 1.03	0.070
Naming	2.14 ± 0.69	2.31 ± 0.81	0.39	1.81 ± 1.06	2.19 ± 0.75	0.137
Memory	3.38 ± 0.86	3.48 ± 0.78	0.63	3.31 ± 0.93	3.81 ± 0.85	0.048^*^
Attention	3.03 ± 0.68	3.21 ± 0.62	0.32	3.69 ± 0.88	4.19 ± 0.85	0.043^*^
Speaking	2.27 ± 0.75	2.48 ± 0.63	0.26	2.42 ± 0.70	2.65 ± 0.56	0.197
Abstract thinking	1.55 ± 0.83	1.59 ± 0.78	0.87	1.00 ± 0.69	1.42 ± 0.81	0.048^*^
Orientation	3.14 ± 1.16	3.59 ± 0.98	0.12	3.19 ± 1.13	3.77 ± 0.95	0.052
MMSE	22.72 ± 1.98	23.86 ± 2.34	0.051	23.12 ± 2.89	25.62 ± 2.35	0.001^*^

^*^P < 0.05.

## 4 Discussion

Revascularization plays a critical role in managing occlusive cerebrovascular conditions; however, no consensus has yet been established regarding the optimal therapeutic strategy for patients with chronic MCAO. despite the use of intensified pharmacological regimens, a considerable proportion of individuals with chronic MCAO continue to experience recurrent TIAs, ischemic strokes, and progressive cognitive decline (14). These outcomes highlight the need for a more proactive approach, with surgical intervention emerging as a potentially beneficial strategy. The present study compared the outcomes of medication alone vs. a combination of medication and interventional surgery (including balloon angioplasty) in a cohort of 55 patients with chronic MCAO. While both groups demonstrated post-treatment improvements in neurological deficits, the interventional group exhibited significantly enhanced cerebral perfusion, as reflected by improvements in CBF and MTT, as well as in cognitive performance, as assessed by the MoCA and MMSE. In contrast, the medication only group did not demonstrate significant changes in either perfusion parameters or cognitive function. These findings indicate that a combined therapeutic approach may provide superior benefits for this population and should be considered as a viable strategy in future treatment planning.

In the context of chronic intracranial arterial occlusion in China, the annual risk of stroke has been reported to exceed 20%, and overall prognosis remains poor (15). Maintaining adequate cerebral perfusion within the affected vascular territories plays a pivotal role in minimizing stroke recurrence and associated mortality. While infarctions in individuals with chronic MCAO are often mild and may respond favorably to pharmacological treatment, frequently resulting in only minor residual neurological deficits or an asymptomatic presentation, neuronal tissue loos due to infarction is generally irreversible. Consequently, persistent sequelae may remain even in cases with limited initial symptomatology. CTP imaging in this population often demonstrates extensive hypo-perfused areas that exceed the size of the infarcted area. These hypo-perfused regions are particularly evident through elevated Tmax and prolonged MTT values, while CBV and CBF may remain within normal ranges due to compensatory collateral circulation. These findings suggest that Tmax and MTT are sensitive indicators of cerebral hypoperfusion and may facilitate early identification of at-risk areas and timely clinical intervention.

Current clinical management often emphasizes the treatment of acute cerebral infarction and its associated symptoms, such as aphasia, motor and sensory impairments, and balance disturbances (16). However, a significant proportion of individuals present with chronic ischemic symptoms, including persistent dizziness and cognitive slowing. Neurocognitive assessments in such individuals frequently reveal measurable cognitive decline. This subgroup is typically affected by progressive atherosclerosis of the MCA, which evolves over an extended disease course, permitting the gradual development of collateral circulation (17). Despite this compensation, infarctions in these individuals often appear as small, scattered, hypo-perfused lesions typically associated with only mild neurological impairment. Despite this relatively benign clinical profile, findings from the present study indicate that cognitive impairment remains prevalent among individuals with chronic MCAO. This observation indicates a potential relationship between prolonged vascular insufficiency and progressive cognitive decline. To date, limited evidence has been available regarding the comparative efficacy of pharmacological vs. interventional approaches in facilitating cognitive recovery in this population. Given that recurrent TIAs and cognitive deterioration may persist despite optimized medical therapy, the integration of interventional procedures into comprehensive treatment planning appears warranted.

Zhao et al. evaluated the safety and efficacy of a combined surgical approach involving superficial temporal artery to middle cerebral artery (STA to MCA) bypass with cranioplasty in individuals with symptomatic chronic internal carotid artery (ICA) occlusion (18). The results indicated that this approach was both safe and effective, contributing to improvements in CBF and relief of clinical symptoms. Additionally, recent findings have affirmed that STA to MCA bypass surgery enhances neurological outcomes in patients with MCAO, particularly among those with insufficient preoperative collateral circulation (19). These findings highlight the therapeutic potential of bypass surgery in the management of chronic intracranial vascular occlusion. However, bypass procedures do not replicate the physiological trajectory of native vascular anatomy or blood flow, which may limit the long-term stability of cerebral perfusion achieved through this method.

In recent years, multiple medical centers have adapted techniques originally developed for the treatment of acute large vessel occlusion to address non-acute symptomatic occlusions. Endovascular recanalization has gained increased attention in such contexts (20). For example, Fude et al. investigated the safety and efficacy of endovascular therapy using balloon angioplasty and stenting (both balloon-expandable and self-expanding) in patients with symptomatic non-acute MCAO. Among 64 patients, successful vascular recanalization was achieved in 57 cases, while seven procedures failed (20). Cagnazzo et al. conducted a feasibility assessment of coronary and carotid artery stent recanalization in five patients with symptomatic hypoperfusion due to chronic ICA occlusion. All participants exhibited sustained reperfusion and symptomatic improvement, thereby demonstrating the potential efficacy of endovascular revascularization in this population (21). These outcomes align with the present findings, further supporting endovascular intervention as a promising strategy for managing chronic intracranial arterial occlusion.

Importantly, Xia et al.'s study mainly assesses the feasibility and safety of endovascular recanalization in non-acute symptomatic MCAO and proposes a new patient classification method, with a focus on the feasibility and safety of the surgical operation itself (8). While Gao et al. analyzed the feasibility and safety of endovascular recanalization, and provides a new angiographic classification, with an emphasis on the treatment evaluation and classification of specific vascular occlusions (14). Different from these studies, our study focuses on the impact of interventional revascularization therapy on cognitive function and cerebral perfusion in patients with chronic MCAO, aiming to explore the role of this treatment in improving the long-term neurological function and cognitive status of patients.

Although revascularization surgery offers a clinically compelling approach for addressing hypoperfusion in high-risk cerebral territories, randomized controlled trials have yet to establish conclusive evidence regarding the safety and efficacy of either surgical or endovascular interventions (22). For instance, Liu Fude et al. reported a failure rate of ~11%, and similar procedural challenges, including instances of recanalization failure and perforator-related complications, were observed in the present study (20). Prior research has emphasized that rigorous patient selection, accurate assessment of procedural indications, and prevention of perioperative complications are essential to reducing long-term stroke risk (23). In addition to demonstrating the safety of interventional surgery, the current findings also underscore its beneficial effect on cognitive function in patients with chronic MCAO. By restoring CBF and regional perfusion, endovascular recanalization may reduce the likelihood of recurrent cerebral ischemia (24).

The patient selection criteria in this study were designed to optimize procedural safety and efficacy. Inclusion was limited to patients with favorable anatomical features, including well-established collateral circulation, adequate visualization of distal vasculature beyond the occlusion via compensatory perfusion, retrograde filling through the anterior cerebral artery and meningeal branches, and occlusion segments of limited length. These factors likely contributed to reduced complication rates. No cases of intraoperative or postoperative hemorrhage were observed, and minor extravasation of contrast agent confined to infarcted regions did not result in worsening of neurological status.

Emerging evidence has identified a relationship between asymptomatic MCAO-related cerebral hypoperfusion and cognitive impairment (25). In regions affected by chronic hypoperfusion, neurons may exist in an energetically compromised but viable state, often referred to as functional dormancy. Restoration of cerebral blood flow via recanalization may reactivate these dormant neurons, thereby enhancing cognitive performance. This pathophysiological mechanism provides a plausible explanation for the improvements in cognitive domains observed in patients following interventional surgery. In the present study, significant improvements were in specific cognitive domains, particularly in attention, abstract reasoning, and memory. Supporting these findings, Kong et al. reported that patients undergoing angioplasty exhibited improved spatial working memory, with regional perfusion levels correlating positively with verbal and visual memory performance (26). In contrast, cognitive function remained unchanged in patients with recurrent stenosis, indicating that improvements in cerebral perfusion are closely associated with memory restoration. Moreover, Zhang et al. identified a link between attention deficits and impaired cerebral perfusion in individuals with depression, although the underlying mechanisms remain incompletely understood (27). Furthermore, a clinical study investigating vascular cognitive impairment demonstrated that increases in cerebral blood velocity following intervention were correlated with improved performance in attention, abstraction, and delayed memory, as well as overall increases in MoCA scores (28). These findings are consistent with the present results, in which cognitive gains were most pronounced in primarily in memory, attention, and abstract reasoning. The potential effects on other cognitive subdomains require further investigation.

In summary, the findings of this study support the therapeutic role of interventional revascularization in improving cognitive function among patients with chronic MCAO. The cognitive enhancements observed in the interventional group appear to be closely associated with improvements in cerebral perfusion. Combined with the favorable safety profile of endovascular procedures demonstrated in this cohort, the results indicate that timely and appropriate surgical intervention may contribute to the mitigation of cognitive decline following cerebral infarction.

However, several limitations must be acknowledged. This study was conducted at a single center, included a relatively small sample size, and featured a follow-up duration limited to 6 months. In addtion, more professional assessment tools should be employed to further evaluate the cognitive status of the patients, such as Clinical Dementia Rating (CDR). Future studies should incorporate multicenter designs with larger cohorts and extended longitudinal follow-up, and introduce more other methods for cognitive assessment to further validate these outcomes.

## 5 Conclusion

Interventional surgery demonstrated superior therapeutic efficacy compared to standardized pharmacological treatment in enhancing cerebral perfusion and cognitive outcomes among patients with chronic MCAO. Cognitive improvements were most evident in the domains of memory, attention, and abstract reasoning. These findings support the integration of interventional revascularization as a potential strategy for optimizing long-term neurological and cognitive recovery in this patient population.

## Data Availability

The raw data supporting the conclusions of this article will be made available by the authors, without undue reservation.
